# Chromatographic fingerprinting of ipratropium and fenoterol in their novel co-formulated inhaler treating major respiratory disorders; application to delivered dose uniformity testing along with greenness and whiteness assessment

**DOI:** 10.1186/s13065-024-01265-5

**Published:** 2024-08-27

**Authors:** Salma N. Ali, Samah S. Saad, Ahmed S. Fayed, Hoda M. Marzouk

**Affiliations:** 1 Pharmaceutical Analytical Chemistry Department, College of Pharmaceutical Sciences and Drug Manufacturing, Misr University for Science & Technology, 6th of October City, Giza, Egypt; 2 Pharmaceutical Analytical Chemistry Department, Faculty of Pharmacy, Cairo University, Kasr Al-Aini Street, Cairo, 11562 Egypt

**Keywords:** AGREE tool, Chronic obstructive pulmonary disease, ComplexGAPI, Delivered dose uniformity, EHS tool, Fenoterol, HPLC–DAD, HPTLC–densitometry, Ipratropium, White analytical chemistry

## Abstract

Ipratropium bromide (IPR) and fenoterol hydrobromide (FEN) have recently been combined in a promising inhaler to treat two prevalent inflammatory illnesses of the airways: bronchial asthma and chronic obstructive pulmonary disease (COPD). The necessity for a single, sensitive, and trustworthy analytical approach to cover the diverse and necessary tests of in-vitro and in-vivo studies is greatly grown with the rising production of new fixed combinations. Two novel, selective and environmentally friendly LC techniques were developed in order to guarantee precise measurement of IPR and FEN in their challenging formulation. The initial technique involved high-performance thin-layer chromatography (HPTLC) in conjunction with densitometric quantification. Chromatographic separation was attained on HPTLC plates utilizing ethyl acetate - ethanol - acetic acid (5.0:5.0:0.1, by volume) as a developing system. Densitometric quantification of the separated bands was carried out at 220.0 nm over concentration ranges of 0.50–15.0 µg/band for IPR and 0.50–12.0 µg/band for FEN. High-performance liquid chromatography (HPLC) paired with diode array detection (DAD) was the core of the second technique. The optimized separation was achieved on a Zorbax SB C_18_ (150 × 4.6 mm, 5 μm) column with a combination of 10.0 mM potassium dihydrogen orthophosphate, pH 5.0 ± 0.1, adjusted with o-phosphoric acid and methanol (70:30, v/v) as the mobile phase and pumped at flow rate of 1.0 mL/min. The peaks were monitored at 220.0 nm using diode array detection, achieving linearity range of 5.0–200.0 µg/mL for both drugs. The ICH criteria have been verified and both methods have been confirmed to be valid, and successfully applied for assay the cited drugs in the Atrovent^®^ comp HFA metered dose inhaler as well as delivered dose uniformity testing of the final product. Finally, whiteness appraisal and several state-of-the-art green evaluation metrics were applied to evaluate the sustainability of the proposed methods. The suggested approaches produced promising results and are the first simple and sustainable methodologies for the simultaneous quantification of both drugs in different real samples, all of which strongly suggest their application in quality control laboratories.

**Supplementary Information:**

The online version contains supplementary material available at 10.1186/s13065-024-01265-5.

## Introduction

Bronchial asthma and chronic obstructive pulmonary disease (COPD) are the two greatest inflammatory diseases of the respiratory tract, and both are increasing worldwide affecting around 600 million people [1]. Bronchodilator treatments given by inhalation are helpful for the symptomatic relief of constriction of the airway in patients with bronchial asthma or COPD in adults and children [2] As a result, by developing a novel medication combination of ipratropium bromide (IPR) and fenoterol hydrobromide (FEN), the pharmaceutical industry has honed its energy to control and manage the symptoms of both COPD and asthma disorders [3].

**Fig. 1:**
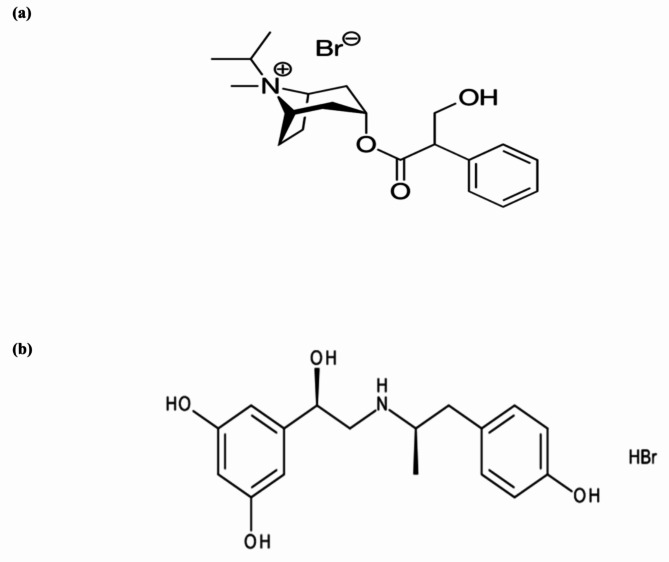
Chemical structures of **(a)** Ipratropium bromide (IPR) and **(b)** Fenoterol hydrobromide (FEN).

IPR, a bronchospasm-related medication approved by the Food and Drug Administration (FDA) [4], is an anticholinergic drug **[**Fig. 1a**]**. FEN is an inhaled bronchodilator medication for asthma. It is a β2-adrenergic agonist **[**Fig. 1b**]** [5]. IPR and FEN should be used together rather than separately for the treatment of acute severe asthma and COPD due to their different mechanisms of action, according to earlier study findings [6–8]. The potential of this combination to increase forced expiratory volume in one second (FEV1) has been studied [9]. Additionally, patients needed less duration using metered dose inhalers (MDI) and experienced effective bronchodilation [6, 10]. On the other hand, taking each drug at a lower dose together could still have the same clinical effect, while potentially decreases the side effects of each drug if administered alone [11].

The literature provided only one HPLC method for determining the drugs under study along with other drugs in various nebulizer solutions [6]. This reported work, in contrast to the proposed research, did not include the analysis of the co-formulated inhaler under study (Atrovent^®^ comp HFA) or delivered dose uniformity testing. Moreover, the guidelines for green chromatography, which protect the environment and ensure analyst safety, were also overlooked. Another study presents an HPLC system to study factors affecting the stability and performance of IPR and FEN pressurized-metered dose inhalers [12], however this procedure was not validated or applicable to dosage form assay.

The priority given to various analytical procedures has changed as a result of the global trend towards environmentally friendly analytical methods depending on how seriously they fulfil the guidelines for green analytical chemistry [13]. The two most widely used and adaptable techniques in the field of pharmaceutical analysis are high performance thin layer chromatography (HPTLC) and high-performance liquid chromatography (HPLC). Both of them provide an automated, quick, simple, and economical method to separate, identify, and quantify challenging mixtures with great repeatability and resolution [14, 15]. Chromatographic techniques have furthermore made major advancements in green analytical chemistry. This was achieved by several attempts that have been undertaken to reduce energy consumption, the usage of hazardous solvents in the mobile phase’s composition and waste production per sample. They facilitated the simultaneous analysis of several analytes [16, 17].

The purpose of this research study was to develop the first simple, precise, and economical HPTLC and HPLC-DAD methods for the simultaneous determination of IPR and FEN in their challenging dosage form. The motivation behind this research was to align with the global trend towards sustainable chemistry and develop a more eco-friendly and safer alternatives. The study also intended to prove that using green analytical techniques in chromatographic separation was achievable without losing analysis parameters. Each of the methods were effectively applied to the metered dosage inhaler and validated according to the ICH guidelines. Following that, a comparative analysis of the stated methods was performed, and their greenness profiles were evaluated *via* several tools; including environmental, health and safety (EHS) tool, Complex Green Analytical Procedure Index (ComplexGAPI), Analytical Greenness metric (AGREE) and White analytical chemistry (WAC).

## Experimental

### Instruments

#### For HPTLC–densitometry

The stationary phase used for the chromatographic separation was HPTLC aluminum sheets (20 × 10 cm) precoated with silica gel 60 F_254_ (Merck, Darmstadt, Germany). Using a 100-µL CAMAG micro-syringe and a CAMAG Linomat 5 autosampler (Muttenz, Switzerland), the samples were dispensed onto the plates. A CAMAG TLC scanner (model 3 S/N 1302319), running with winCATS software (Muttenz, Switerland) was used for scanning and densitometric analysis. With reflectance measuring mode and a 20.0 mm/s scanning speed, the slit dimension was set to 3.0 × 0.45 mm. The radiation source used came from a deuterium lamp.

#### For HPLC–DAD

The HPLC equipment used in the experiment was an Agilent^®^ 1260 HPLC separations module (Milford, United States), which included an auto-sampler, degasser, quaternary pump, column compartment, and photodiode array detector (DAD). Agilent^®^ chemStation software was employed to process and modify data. A Zorbax^®^ SB C_18_ column (150 × 4.6 mm, 5 μm) from GL Sciences (Barcelona, Spain), was employed. The pH was adjusted using a Jenway^®^ pH meter (model 3510) from (Felsted, Essex, UK).

### Reagents and materials

#### Pure samples

Pure standard IPR and FEN were kindly provided by the Global Napi Pharmaceuticals (GNP) Company (Al-Giza, Egypt). According to their BP official methods [18], their potency was checked and found to be 99.40% ± 1.062 and 99.40% ± 0.926 for IPR and FEN, respectively.

#### Pharmaceutical formulation

Atrovent ^®^ comp HFA (Batch No. 104604), each metered dose is labeled to contain 20.0 µg IPR and 50.0 µg FEN, manufactured by Boehringer Ingelheim. It was bought from Egyptian drugstore.

#### Chemicals and solvents

Ethyl acetate, ethanol, and glacial acetic acid (Pioneer Chemical Co., Giza, Egypt) were all used as analytical-grade chemicals and solvents, Potassium dihydrogen orthophosphate (Sigma-Aldrich, Steinheim, Germany). Also, methanol of HPLC -grade (Fisher Scientific, UK) was used.

#### Standard solutions

To prepare individual stock solutions with a concentration of 1.0 mg/mL of IPR and FEN, 10.0 mg of each standard material was precisely weighed and put into separate 10-mL volumetric flasks. The materials were dissolved in methanol and then diluted with methanol. By methanol dilution of the stock solutions, working standard solutions were prepared to achieve a concentration of 200.0 µg/mL. It was found that stock solutions for IPR and FEN can be stored stable for up to a week at 4^°^C in the refrigerator with light protection.

### Procedures

#### Chromatographic conditions

##### For HPTLC–densitometry

Each sample was applied separately on HPTLC aluminum plates (20 × 10 cm) using a 100-µL micro-syringe with autosampler. The samples were placed 10.0 mm from the plates’ bottom border and the sides in bands that were 6.0 mm broad. To accomplish the separation, 60.0 mL of the developing system, composed of ethyl acetate – ethanol - acetic acid in a ratio of 5.0:5.0:0.1, by volume, was placed within a binary glass chamber. This developing system was left in the chamber for saturation at room temperature (25.0 ± 2^°^C) for 30.0 min. After that, the plates were allowed to develop vertically, reaching a distance of 8.0 cm in a linear ascending direction. Following the developing procedure, the plates were then removed and dried with air. They were densitometrically scanned at a measuring wavelength of 220.0 nm. The scanning speed was set at 20.0 mm/s. Densitograms, and integrated peak areas were the results of the scanning process.

##### For HPLC–DAD

Separation was performed at room temperature using a Zorbax^®^ SB C_18_ column (150 × 4.6 mm, 5 μm). The mobile phase composed of two solvents: the first, is 10.0 mM potassium dihydrogen orthophosphate with pH adjusted to 5.0 ± 0.1 by o-phosphoric acid, and the other solvent is methanol in ratio of 70:30, v/v. Solvents were degassed in an ultrasonic bath for 10.0 min and passed through a 0.45-µm Millipore membrane filter before use. The mobile phase was pushed through the column at a rate of 1.0 mL/min during the isocratic elution process. After filtration, samples were injected into the HPLC system in 10-µL quantities using an autosampler. The separated peaks were detected and quantified at a wavelength of 220.0 nm.

#### Linearity

##### For HPTLC–densitometry

Using the HPTLC system, bands were applied using accurately measured aliquots of the relevant IPR (0.5, 1.0, 2.0, 5.0, 10.0 and 15.0 µL) and FEN (0.5, 2.0, 5.0, 8.0, 10.0 and 12.0 µL) stock standard solutions in the range of 0.5–15.0 and 0.5–12.0 µg/band, respectively. The procedure was carried out under the previously described chromatographic conditions. Calibration curves were generated by collecting scanning profiles, to plot the average integrated peak area against the relevant drug concentration where polynomial regression equations were computed.

##### For HPLC–DAD

Using the mobile phase as a diluent, linearity ranges were determined through preparing serial dilutions of each drug, separately, with concentration ranges of 5.0–200.0 µg/mL for IPR and FEN. Then, after injecting each of the prepared solutions in triplicates, chromatogram for each sample was obtained. The chromatographic conditions that were previously mentioned were used, and the peak areas were integrated. Following that, calibration curves were constructed to establish the correlation between the average integrated peak areas and its respective concentrations, and linear regression equations were demonstrated.

#### Analysis of laboratory-prepared mixtures

Aliquot quantities from stock standard solutions of the investigated drugs have been mixed in various ratios, both above and below the branded ratio labeled in the dosage form. This was performed to construct synthetic binary mixtures for analysis. The samples were dissolved and diluted with methanol or mobile phase to the proper concentrations in 10-mL measuring flasks, and then analyzed under the aforementioned chromatographic conditions applicable to both methods.

#### Application to pharmaceutical formulation

Accurately 2.0 mL of the metered dose inhaler solution (Atrovent^®^ comp HFA), equivalent to 800.0 µg IPR and 2000.0 µg FEN, were transferred in 10-mL volumetric flasks. They were mixed with methanol and diluted to reach the final concentrations of 0.08 and 0.20 mg/mL, respectively. Appropriate dilutions with methanol or mobile phase were applied to obtain concentrations within the linearity ranges for each of the determined analytes. Both methods applied using the previously described chromatographic system. The respective regression equation was used to calculate the recovery percent (R%) and the concentrations of the investigated drugs. Furthermore, the standard addition technique was applied. This involved spiking exact quantities of standard IPR and FEN to Atrovent^®^ comp HFA nebulizer solutions and completing the mixtures with methanol or mobile phase. The samples were then analyzed as previously described.

#### Testing the delivered dose uniformity

In compliance with international guidelines [19, 20], the proposed HPLC-DAD method was additionally applied to evaluate the delivered dose uniformity of the marketed combined inhaler. IPR and FEN content in Atrovent^®^ comp HFA inhaler were determined using the same procedure as previously described, with the exception that just two actuations (two dosage unit) were applied to a 5-mL volumetric flask and diluted with mobile phase to the flask mark. Two distinct groups were used for this study from two different batches of metered dose inhalers (GP1; B.N. 104604, GP2; B.N. 204632), each group contained 10 units. For each group, the procedure was repeated ten times, each time with a new delivered dosage unit.

## Results and discussion

In quality control laboratories, where efficiency and cost are critical, the release of new pharmaceutical formulations requires the use of straightforward, precise, affordable, and quick analytical techniques. Developing a safer methodology presents a challenge in striking a balance between reducing toxicity and preserving the efficacy of the method, (i.e.) eco-friendly approaches have evolved as an attractive strategy [21]. This study is a more environmentally friendly option compared to other complicated chromatographic procedures since it requires few-to-no tedious sample preparations and considered to be straightforward, automated, and highly reproducible. The concurrent analysis of multiple samples simultaneously conserves environmental resources by drastically reducing the amount of energy and solvents used for each sample [22, 23]. Thus, the goal of this work is to develop the first chromatographic techniques that are simple, selective, and eco-friendly for the simultaneous quantification of IPR and FEN in their challenging inhaler dosage form.

### Method development and optimization

#### For HPTLC–densitometry

**Fig. 2:**
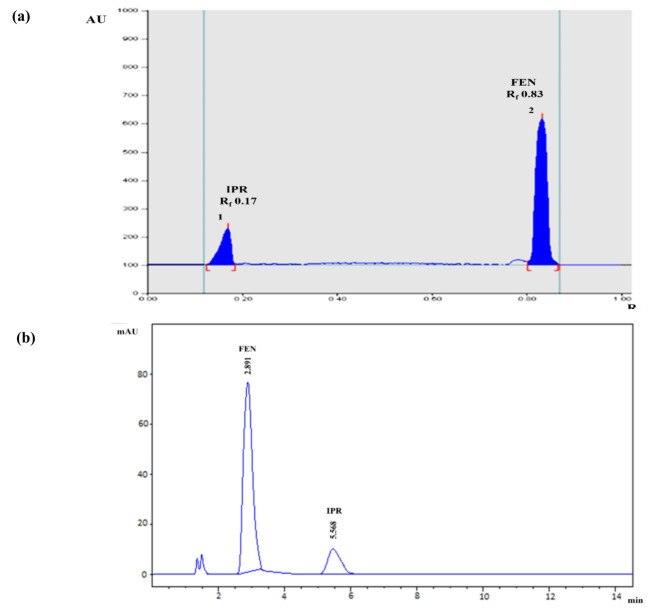
**(a)** HPTLC densitogram of resolved mixture of IPR and FEN (6.0 µg/band, each) and **(b)** HPLC-DAD chromatogram of resolved mixture of IPR and FEN (70.0 µg/mL, each)

The evaluation of several developing systems with diverse compositions and ratios served as the foundation for the method development. To avoid their hazardous nature and adverse environmental effects, chloroform, toluene, and benzene were excluded from the trials. Several trials were conducted to optimize the mobile phase composition and scanning wavelength with the aim to reach optimum separation between the drugs under study and establish symmetrical peaks with appropriate R_f_ values. Different eco-friendly solvent systems, such as ethyl acetate-ethanol and ethyl acetate- methanol, were examined in different ratios (5:5, 4:6, 6:4, 7:3 and 8:2, v/v) for acceptable separation performance. For the ethyl acetate-methanol system, FEN band displayed a high R_f_ value above 0.6, while the other band of IPR did not move away from the baseline. Using ethyl acetate-ethanol system, poor separation of the investigated compounds and tailed peaks were shown. The experimental results were not efficient. Furthermore, various small quantities of acetic acid, formic acid, or aqueous ammonium hydroxide solution were evaluated as potential additives to the ethyl acetate-ethanol system. Initial results with ammonia and formic acid showed poor resolution with tailed peaks. The addition of small amounts of acetic acid to ethyl acetate-ethanol system achieved the desired outcomes; the shape of peaks was improved, and peak tailing problem was solved, while resolution was satisfactory. The use of ethyl acetate - ethanol - acetic acid with different ratios was investigated and adjusted to provide the highest resolution of the binary mixture and the optimum chromatographic separation; the system of choice was ethyl acetate-ethanol-acetic acid (5.0:5.0:0.1, by volume), Fig. 2a. Several wavelengths (210.0, 220.0, 250.0, and 275.0 nm) were considered for densitometric measurements; the one with the highest sensitivity for each component with minimum noise was 220.0 nm.

#### For HPLC–DAD

The method was developed to be eco-friendly by avoiding the use and generation of harmful chemicals, minimizing waste, and providing a quick analysis time. By this way, routine determination of IPR and FEN determination could be achieved without causing harm to the environment. Primarily, mixtures of methanol-water and methanol-acetate buffer were tested in different ratios at different pH values (4.0–6.0); but the resolution was poor. After that, different methanol-potassium dihydrogen orthophosphate buffer ratios (with several pH values, 3.0–7.0) were tested by applying isocratic elution for studying the parameters influencing chromatographic separation performance. A 10.0 mM potassium dihydrogen orthophosphate at pH 5.0 ± 0.1, and methanol were used in different ratios to provide reasonable results. It was observed that excellent separation was achieved upon raising the buffer ratio. Several stationary phase columns, including C_8_ and C_18_ in different dimensions, were carefully examined. The tried C_8_ columns displayed non-resolved solvent and FEN peaks. By substituting the C_18_ column for the C_8_ column, the separation was enhanced and the optimal resolution for the analytes was achieved. The best resolution was obtained using Zorbax^®^ SB C_18_ column (150 × 4.6 mm, 5 μm). Different wavelengths (210.0, 220.0 and 275.0 nm) were considered for DAD measurements. The UV-absorption spectra of IPR and FEN were recorded in the range of 200.0–400.0 nm and were utilized for choosing the optimum wavelength for detection, Fig. S1. The selection criterion favored IPR because of being lower constituent in the dosage form ratio, which led to wavelength choice of 220.0 nm. Additionally, the IPR spectrum shows low absorptivity at higher wavelengths. Furthermore, FEN, the synchronized drug, exhibit high absorbance at the selected wavelength, enabling the highest level of sensitivity for both drugs. Optimal separation of the binary mixture was achieved using an isocratic mobile phase composed of 10.0 mM potassium dihydrogen orthophosphate (pH 5.0 ± 0.1, adjusted by adding o-phosphoric acid) and methanol (70:30, v/v) with flow at a rate of 1.0 mL/min. The column used for this method was Zorbax^®^ SB C_18_ (150 × 4.6 mm, 5 μm). It provided a satisfactory separation of the two peaks in a short run time within 7 min. It demonstrated high sensitivity for the investigated substances. Figure 2b shows a chromatogram with the separated peaks with acceptable resolution and an appropriate analysis time.

### System suitability parameters

System suitability parameters were monitored to ensure the performance of the two optimized chromatographic systems. For the HPTLC-densitometry technique, they included retardation factor, capacity factor, tailing factor, resolution and selectivity [24], that were calculated and provided satisfactory results for both drugs. Suitability parameters were also calculated for the HPLC-DAD method. The acquired outcomes match up with the acceptance values [25, 26] proving that the proposed method functioned well. Results obtained and data for both methods are tabulated in Table 1.

**Table 1:** System suitability parameters of the proposed HPTLC–densitometric and HPLC–DAD methods for the determination of Ipratropium and Fenoterol

Method	Parameters	IPR	FEN	Reference value [24]
**HPTLC-densitometry**	**Retardation factor (** ***R***_***f***_ **)** **± 0.02** ^**a**^	0.17	0.83	
	**Capacity factor (** ***k*** ^***’***^ **)** ^**b**^	4.88	0.20	
	**Selectivity factor (** ***α*** **)** ^**c**^	24.40		α > 1
	**Resolution (** ***Rs*** **)** ^**d**^	14.88		R_s_ > 1.5
	**Tailing factor (** ***T*** **)** ^**e**^	0.83	1.00	T ≤ 2
**Method**	**Parameters**	**FEN**	**IPR**	**Reference value** [26]
**HPLC-DAD**	**Retention time (** ***t***_***R***_ **) (min ± 0.1)**	2.89	5.56	
	**Selectivity factor (** ***α*** **)** ^**c**^	2.86		***α*** > 1
	**Resolution (** ***Rs*** **)** ^**d**^	3.43		R_s_ > 2
	**Tailing factor (** ***T*** **)** ^**e**^	1.10	1.00	T ≤ 2
	**Column efficiency (N)** ^**f**^	2216	3555.45	*N* > 2000
	**Height equivalent to theoretical plate (mm/plate)** ^**g**^	0.067	0.042	

^a^ Retardation factor (*R*_*f*_) = distance traveled by the analyte/distance traveled by the solvent front

^b^ Capacity factor (*k’*); *k’* = (1 – R_*f*_)/R_*f*_ for HPTLC and *k´* = (t_R_ - t_0_) / t_0_ for HPLC.

^c^ Selectivity (*α*) = k’_2_/k’_1,_ calculated for each of two successive peaks

^d^ Resolution(*Rs*); *Rs* = R_*f*2_ – R_*f*1_/0.5 (w_1_ + w_2_), where R_*f*_ is the retardation factor and w is the peak width calculated for each of two successive peaks for HPTLC and *Rs* = [2 (t_R2_ - t_R1_)] / (W_1_ + W_2_) for HPLC.

^e^ T = W_0.05_/2f, where W_0.05_ is the width of the peak at 5% height and f is the distance from peak maximum to the leading edge of peak

^f^*N* = 16 (t_R_ / w)^2^, where w is the peak width

^g^ HETP = L /N, where L is the column length (mm)

### Method validation

The ICH guidelines for the validation of analytical procedures [27] were implemented to evaluate the proposed chromatographic methods’ validation criteria.

#### Linearity and concentration ranges

For HPTLC-densitometric method, calibration curves were produced with polynomial regression equation, displaying the correlation between the integrated peak area and the corresponding concentrations within the ranges of 0.5–15.0 and 0.5–12.0 µg/band of IPR and FEN, respectively, **Fig. S2**. While the proposed HPLC-DAD method possessed linear correlations in a range of 5.0–200.0 µg/mL for both drugs. Table 2 displays the complete parameters of the regression equations for each method.

**Table 2:** Method validation parameters for determination of Ipratropium and Fenoterol by proposed chromatographic methods

Method Parameter	HPTLC–densitometry		HPLC-DAD	
	IPR	FEN	IPR	FEN
**Linearity Range**	0.50–15.0 µg/band	0.50–12.0 µg/band	5.0-200.0 µg/mL	5.0-200.0 µg/mL
**Regression equation parameters**				
**Slope (b)** ^**a**^	---	---	4.273	20.418
**Coefficient 1 (b1)** ^**b**^	-9.36	-94.86	---	---
**Coefficient 2 (b2)** ^**b**^	670.27	2141.50	---	---
**Intercept (a)** ^**a, b**^	162.17	217.83	-5.899	11.208
**Correlation coefficient (r)**	0.9999	0.9999	0.9999	0.9999
**Accuracy (mean ± SD)** ^**c**^	100.13 ± 1.608	99.15 ± 1.215	99.85 ± 1.218	100.10 ± 0.786
**Precision (±%RSD)**				
**- Repeatability** ^**d**^	0.786	0.671	0.385	0.488
**- Intermediate precision** ^**e**^	0.944	0.838	0.564	0.678
**Specificity (mean ±%RSD)**	100.15 ± 1.185	100.74 ± 0.945	99.95 ± 1.214	101.07 ± 0.239
**LOD** ^**f**^	0.145	0.151	1.593	1.515
**LOQ** ^**f**^	0.439	0.459	4.828	4.591
**Robustness (RSD%)** ^**g**^	1.105	1.820	1.294	0.697

^a^ Regression equation for HPLC: A = a + bc, where ‘A’ is the average peak area and ‘c’ is the concentration (µg/mL)

^b^ Coefficients 1 and 2 are the coefficients of X^2^ and X, respectively. Following a polynomial regression: A = b_1_ × ^2^ + b_2_x + a, where ‘A’ is the average peak area, ‘c’ is the concentration (µg/band), ‘b_1_’ and ‘b_2_’ are coefficients 1 and 2, respectively and ‘a’ is the intercept

^c^ Accuracy [average of five different concentrations of three replicates each (*n* = 15)]

^d^ Intra-day precision [average of three different concentrations of three replicates each (*n* = 9) within the same day]

^e^ Inter-day precision [average of three different concentration of three replicates each (*n* = 9) repeated on three successive days]

^f^ LOD and LOQ are calculated from the standard deviation (SD) of the y-intercepts and the slope of calibration curve (S) as follows: LOD = 3.3 × SD / S and LOQ = 10 × SD / S

^g^ For HPTLC: average of the change in ethyl acetate ratio (± 1%), developing distance (± 0.5 cm) and scanning wavelength (± 1 nm). For HPLC: average for the change in phosphate buffer ratio (± 1%), flow rate (± 0.1 mL/min) and buffer pH (± 0.1)

#### Accuracy and precision

Using the optimal chromatographic conditions, five individual pure samples of IPR and FEN were examined to evaluate the accuracy of the suggested methods. The regression equation relevant to each drug was used to calculate its concentrations. As displayed in Table 2, the accuracy of the proposed methods was validated by achieving the acceptable mean percentage recoveries. The assay of three various concentrations of each drug was repeated three times on the same day and three consecutive days to assess the intra- and inter-day precisions, respectively. After calculating the relative standard deviation (%), outcomes are shown in Table 2.

#### Selectivity and specificity

To evaluate method selectivity for both methods, laboratory binary mixtures for IPR and FEN were prepared in different concentrations within the aforementioned linearity ranges. These mixtures had different ratios that were both above and below the claimed label of the dosage forms. Using the previously defined methods, several laboratory admixtures were quantified. Table 2 gives an impression about the analysis results, including mean percentage recoveries and percentage relative standard deviation (RSD%) values. Detailed results are displayed in Table S1.These results point out satisfactory selectivity. The complete separation of the binary mixture containing the two drugs under study, IPR and FEN, confirmed the specificity of the two proposed methods, Fig. 2. Furthermore, no additional peaks or interferences from the common excipients or additives in the dosage form were observed, **Fig. S3.** In order to assess the purity of the resolved peaks from any co-eluting interferents, the work also made effective use of DAD for recording UV absorption spectra at multiple points across IPR and FEN peaks. **Fig. S4** illustrates how the peaks of the two drugs were colored green, and all scan paths (represented by black diamonds) fell within the green zone at 990 peak threshold values [28–30]. Peak purity factors for IPR and FEN were 999.934 and 999.973, respectively. Furthermore, specificity was verified by examining the blank sample chromatogram, as shown in Fig. S5.

#### Limit of detection and quantification

The slope of calibration curves and the standard deviation of y-intercepts of the regression lines were used for calculating LOD and LOQ, Table 2. The proposed methods exhibit good sensitivity, as demonstrated by the low LOD and LOQ values obtained.

#### Robustness

The ethyl acetate ratio (± 1%), developing distance (± 0.5 cm), and scanning wavelength (± 1.0 nm) changes were averaged for HPTLC, whereas the phosphate buffer ratio (± 1%), flow rate (± 0.1 mL/min), and buffer pH (± 0.1) changes were averaged for HPLC-DAD. All of the outcomes fell within the allowable range. The robustness of the proposed methods was confirmed by ensuring that the validation parameters were maintained within an acceptable range and the pooled relative standard deviation (%) was less than 2%, Table 2.

### Analysis of the pharmaceutical formulation (atrovent ^®^ comp HFA)

The Atrovent^®^ comp HFA metered dose inhaler was successfully analyzed using the proposed HPTLC and HPLC systems, confirming the absence of excipient interference. IPR and FEN in their dosage forms have been selected. Furthermore, insurance of the validity of the proposed methods was shown through the application of a standard addition technique. The outcomes are displayed in Table 3.

**Table 3:** Quantitative estimation of Ipratropium and Fenoterol in Atrovent^®^ comp HFA inhaler solution and application of standard addition technique

Pharmaceutical formulation	HPTLC-densitometry					HPLC-DAD				
**Atrovent** ^®^ **comp HFA** (Each metered dose is labeled to contain 20.0 µg IPR and 50.0 µg FEN) )B.N. 104604)	**Drug**	**%Found ± SD** ^*^	**Standard Addition Technique**			**Drug**	**%Found** **± SD** ^*^	Standard Addition Technique		
			**Claimed** **(µg/band)**	**Pure added** **(µg/band)**	**%Recovery of the pure added** **amount** ^*^			**Claimed** **(µg/mL)**	**Pure added** **(µg/mL)**	%Recovery of the pure added amount ^*^
	**IPR**	100.78 ± 1.003	**1.6**	0.8	100.93	**IPR**	100.31 ± 1.533	**20**	10.0	100.59
				1.6	99.47				20.0	98.90
				3.2	99.71				40.0	99.27
			**Mean ± SD**		**100.04** **± 0.783**			**Mean ± SD**		**99.59** **±** **0.888**
	**FEN**	100.03 ± 1.121	**4**	2.0	99.34	**FEN**	100.04 ± 0.847	**50**	25.0	100.94
				4.0	101.73				50.0	100.40
				8.0	99.74				100.0	98.75
			**Mean ± SD**		**100.27 ± 1.280**			**Mean ± SD**		**100.03** **±** **1.141**

^*^ Average of five determinations

### Evaluation of the delivered dose uniformity

For the quality control study of the final product of the metered dose inhaler, delivered dose uniformity test was applied to Atrovent ^®^ comp HFA metered dose inhaler [31]. Using the HPLC-DAD technique, ten units for each group were individually analyzed in accordance with the international guidelines [19, 20], to track the uniformity of delivered dosage units. The following formula was used to calculate the dosage form acceptance value (AV) for each group [32].

AV = |M − X̄ | + ks.

**Fig. 3:**
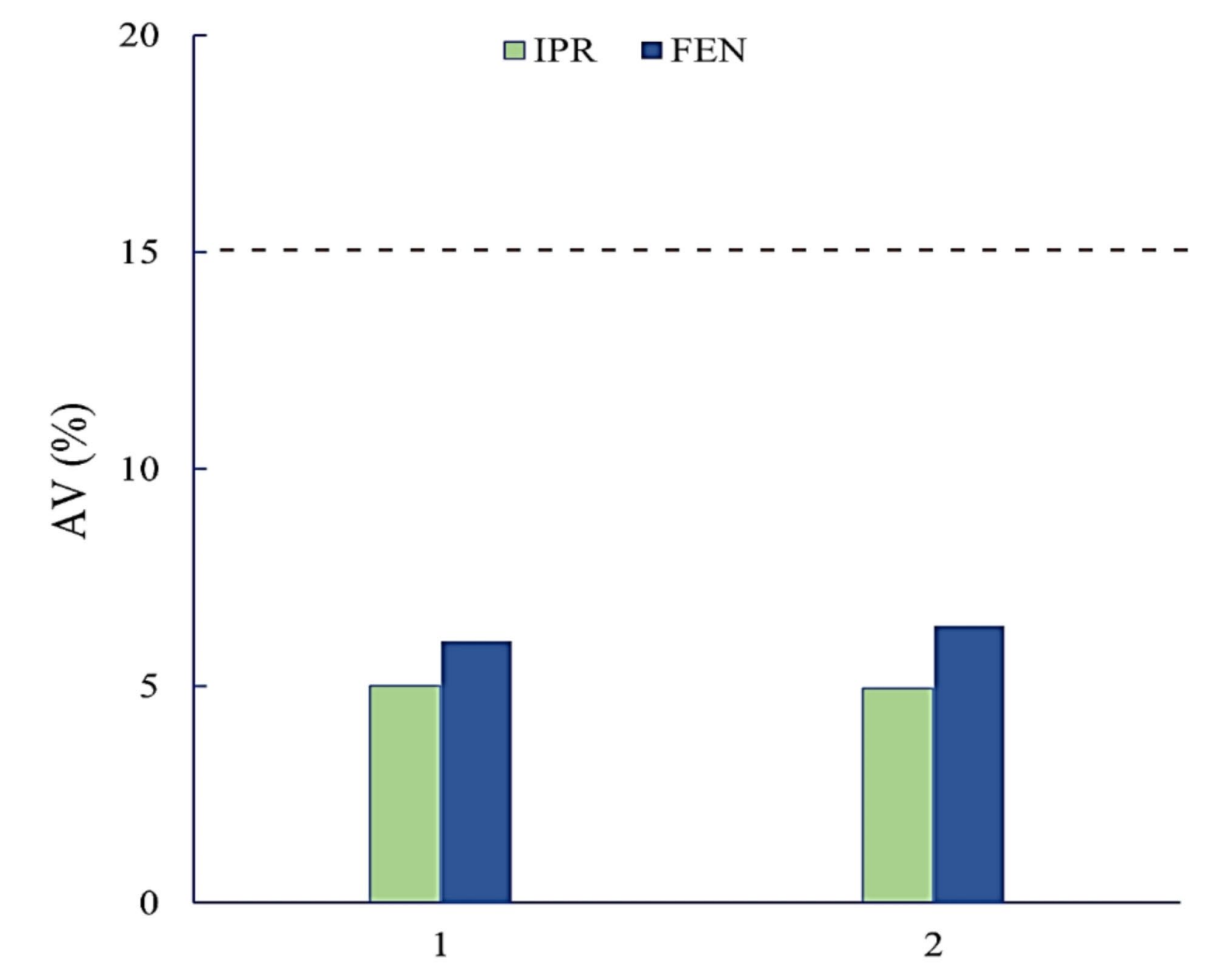
Calculated AV values of IPR and FEN for group 1 (inhaler 1; B.N. 104604) and group 2 (inhaler 2; B.N. 204632) in Atrovent^®^ comp HFA oral inhaler. Each group of 10 units was individually assayed. The dashed line indicates the requirements of the USP Pharmacopoeia (AV ≤ 15%).

Where ‘AV’ represents the acceptance value, ‘M’ is the reference value which equals ‘X̄’ (if 98.5% ≤ X̄ ≤101.5%); it may also be equal to 98.5% or 101.5% (if X̄ <98.5% or X̄ >101.5%), respectively, ‘X̄’ is the mean recovery percent for the assayed ten dosage units (two actuations each), ‘k’ is the acceptance constant which equals 2.4 for ten units, and ‘s’ is the standard deviation of the units. If the ‘AV’ of the 10 dose units is less than or equal to 15.0%, the dosage uniformity requirements are satisfied. The calculated ‘AV’ values are presented in Table 4. As shown in Fig. 3, the ‘AV’ values for each group analyzed by the proposed HPLC method were less than the maximum allowed acceptance value (L1) of 15, proving an acceptable level of delivered dosage uniformity.

**Table 4:** Results of delivered dose uniformity testing for determining Ipratropium and Fenoterol in Atrovent^®^ comp HFA using the proposed HPLC method

Atrovent^®^ comp HFA meter dose no.	Label claim (%)				
	Group 1 (Inhaler 1; B.*N*. 104604)			Group 2 (Inhaler 2; B.*N*. 204632)	
	IPR	FEN		IPR	FEN
**1**	100.42	98.85		100.49	103.48
**2**	97.37	103.89		99.73	97.78
**3**	97.52	104.56		103.41	103.48
**4**	100.76	104.51		102.73	103.74
**5**	100.60	100.37		100.68	104.18
**6**	103.20	103.79		98.21	103.94
**7**	101.38	101.13		100.69	103.61
**8**	102.62	99.07		101.55	103.90
**9**	103.07	102.65		102.35	99.76
**10**	102.24	103.49		105.45	103.33
**Mean**	100.92	102.23		101.53	102.72
**SD**	2.09	2.20		2.05	2.15
**RSD%**	2.07	2.15		2.02	2.09
**AV** ^*^	5.01	6.03		4.95	6.38

^*^ Acceptance value = |M − X̄ | + 2.4 × SD with maximum allowed level (L1) is 15

### Greenness profile assessment

In 2000, the term “Green Analytical Chemistry” (GAC) emerged to reduce the negative effects that methods of analysis have on the environment and human health [33]. It has become crucial to strike a compromise between obtaining results of highest quality and reducing the environmental risks caused by methods of analysis. Due to their potentially harmful effects on human health and the environment, selecting a solvent is one of the most important steps in developing a method. Ethyl acetate and ethanol are recommended as green solvents by the solvent sustainability guide, which also color-codes acetic acid and methanol yellow due to their limitation [34, 35]. Additionally, the three tools listed below were used to assess and confirm the level of greenness for the proposed methods, namely, the Environmental, Health, and Safety (EHS) tool, the Analytical Greenness metric (AGREE) and the Complementary Green Analytical Procedure Index (ComplexGAPI).

#### Environmental, Health, and Safety (EHS) tool

**Fig. 4:**
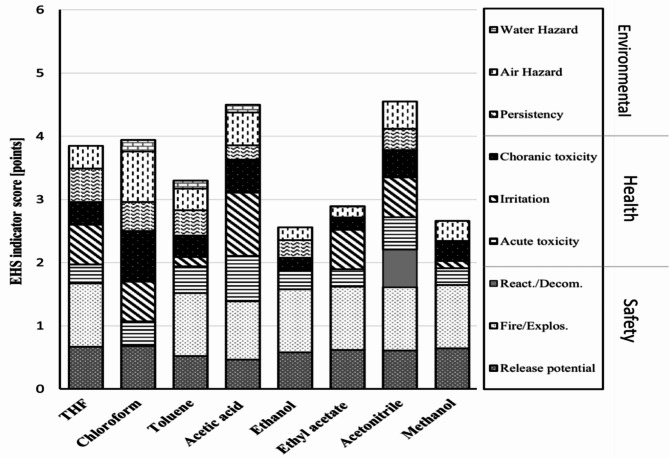
EHS assessment for different solvents used in the two proposed chromatographic methods and reported method [6]

Koller [36] has developed the environmental, health, and safety (EHS) tool for the quantitative screening of potential solvent hazards. This regarded to be a helpful tool for assessing different solvents using nine hazardous categories organized into three sets; the lower the score (closer to 0), the more environmentally friendly the solvent would get [37]. As shown by the EHS graph in Fig. 4, the solvents utilized in the proposed methods; ethyl acetate and ethanol in HPTLC, and methanol in HPLC are considered to be the greener solvent when compared to other commonly used solvents in HPTLC method, such as chloroform and toluene, and the commonly used solvent in HPLC method, acetonitrile. Also, the EHS graph shows preference for the solvents used in proposed chromatographic methods in comparison to that used in reported HPLC [6] method.

#### Complex Green Analytical Procedure Index (ComplexGAPI)

**Fig. 5:**
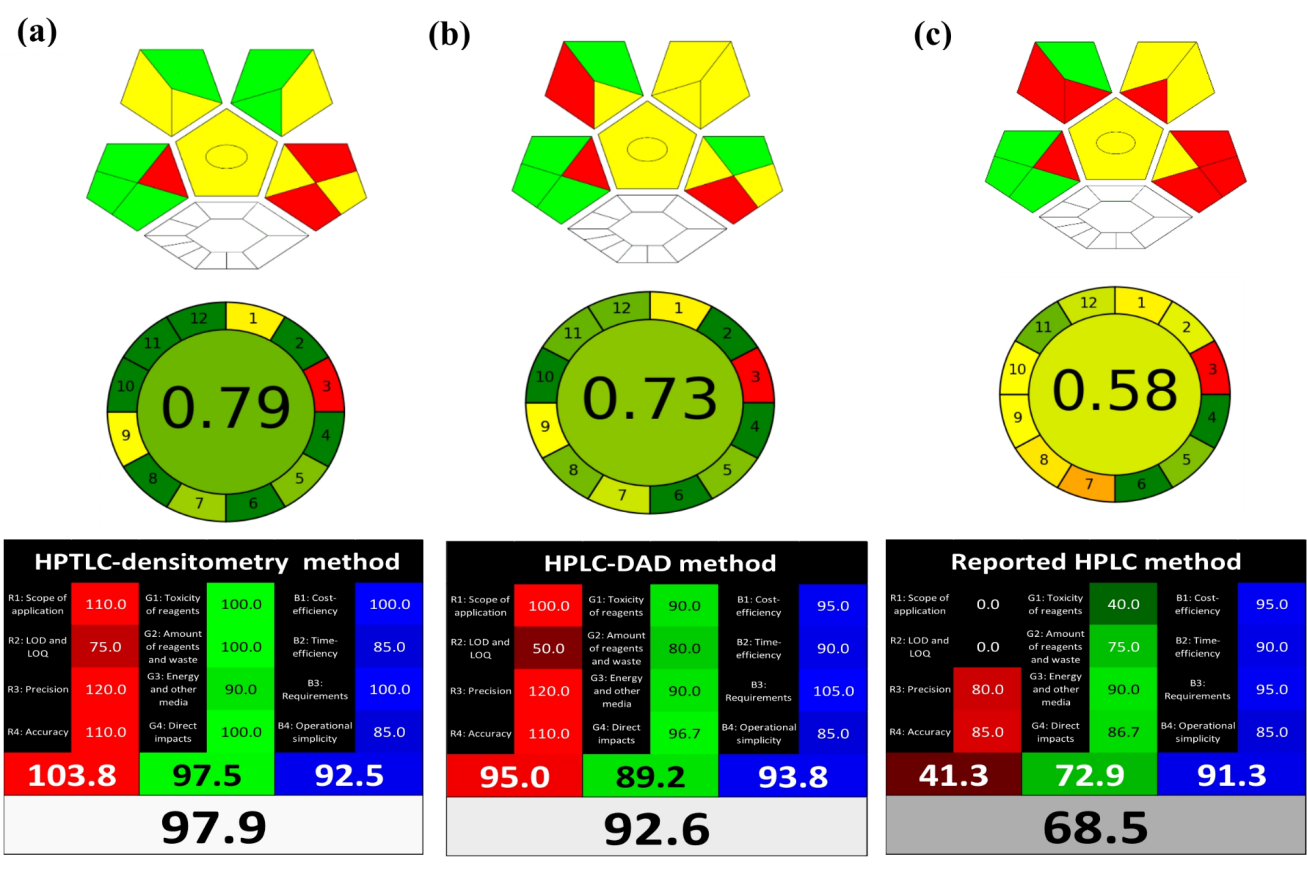
Greenness profile and whiteness assessment of the proposed **(a)** HPTLC-densitometry, **(b)** HPLC-DAD and **(c)** the reported HPLC [6] methods via ComplexGAPI, AGREE and RGB12 whiteness tool

This is a new and simple tool that improves the original GAPI metric. The pictogram for GAPI, with five pentagrams, is expanded by a hexagonal field at the bottom in the ComplexGAPI metric. This field reflects how “green” pre-analysis procedures are. ComplexGAPI deals with all steps of the pre-analysis and analysis process. The modified tool uses a color scale, just like in GAPI, with two or three levels of assessment for each step. From green to yellow to red, the generated pictogram can be used to assess and quantify the low, medium, and high environment impact for each step, respectively. The several aspects of the described processes and analytical protocol are represented by different fields. If certain requirements are met, these fields are filled in green [38]. By looking at the obtained pictograms in Fig. 5, HPTLC pictogram is greener than the HPLC-DAD pictogram, and both show less red-shaded sections, indicating green analytical methods. Upon comparing the proposed chromatographic methods with the published HPLC method [6], the green color of the proposed methods was more prominent because the used solvents have the highest level of greenness in addition to using less waste per sample analysis as shown in Fig. 5.The hexagonal field in the bottom with no colors, due to all methods have no pre-analysis processes.

#### Analytical GREEnness metric (AGREE)

It is an advanced software for evaluating greenness [39]. A fraction of one, ranging from zero to one, is the final score in AGREE. Twelve sections make up the automatically generated pictogram. The color of each section ranges from deep green (1) to deep red (0). The center of the circular pictogram contains the final score. Basic principles like inclusivity, simplicity, flexibility in input and output clarity were taken into consideration [40]. Figure 5 presented that both proposed methods show AGREE pictograms with only one red zone, which correspond to off-line sampling. The overall score shown in HPTLC pictogram (0.79) is higher than HPLC one (0.73). But in general, overall score for both proposed chromatographic methods indicate the highest ecological compatibility and lowest negative impacts of these methods when compared to the reported HPLC [6] method with overall score (0.58), respectively, as shown in Fig. 5.

### White analytical chemistry (WAC)

The goal of the white analytical chemistry method is to provide a distinct tool for applying sustainable development concepts in analytical chemistry [41–43]. These principles are composed of three complementary sections, each of which is colored differently (red, green, or blue). These sections evaluate distinct concepts related to the analytical method. Together, the aforementioned colors combine to produce the white color of the method. In these pillars, the analytical method efficacy is evaluated in the red section, the environmental impact is evaluated in the green section, and the practical usefulness and economic conditions are simply evaluated in the blue section. The WAC tool is also referred to as RGB 12 depending on the number of rules involved. “White” is defined by the WAC as a purpose-driven, well-matched analytical approach [44, 45]. The recommended chromatographic methods were investigated and objectively compared with each other and with the HPLC reported method [6]. Figure 5 reports the evaluation results and the arithmetic mean values for each of the three bands - R(%), G(%), and B(%) of the proposed method using the WAC tool. The overall score of 97.9%, it was determined that the prescribed HPTLC-densitometry performed better than the proposed HPLC-DAD, which came in second place with 92.6%. Reported HPLC method came in third place with overall score of 68.5%, as displayed in Fig. 5. The enhanced analytical performance of the proposed HPTLC-densitometry and HPLC-DAD methods over the published HPLC method for the assay of the marketed dosage form was illustrated by the RGB12 algorithm tool in Fig. 5. Additionally, the ability of HPLC-DAD method to test delivered dose uniformity expands its scope of application. Because of their ease of use and green credentials, the proposed methods were found to be more functional and sustainable.

### Statistical analysis and methods ‘evaluation

A statistical comparison was performed between the results of the IPR and FEN analyses in pure forms as obtained by the proposed methods, and the results obtained from their approved potentiometric titration and titrimetric methods, respectively [18]. The t-test and F-test results showed that there was not a significant difference in terms of accuracy and precision between the proposed and official methods, **Table S2**.

The proposed methods outperform the previously published HPLC method in terms of applicability, cost-effectiveness, efficiency, and ecological sustainability when it comes to the simultaneous assay of FEN and IPR in their metered dose inhaler dosage form. Additionally, the delivered dose uniformity testing was performed by the proposed HPLC-DAD method to evaluate the final product’s quality control, as illustrated in **Table S3**.

## Conclusion

This work provided a successful attempt to develop new, selective and versatile HPTLC–densitometric and HPLC–DAD methods for the simultaneous determination of IPR and FEN in their challenging pharmaceutical co-formulated inhaler treating major respiratory disorders. Moreover, the higher sensitivity features of the developed HPLC-DAD method was exploited for testing delivered dose uniformity of IPR and FEN in Atrovent ^®^ comp HFA metered-dose inhaler. The concept of sustainable development has recently gained power among analytical laboratories and instrumental corporations. A lot of attention was given to using safer and less harmful solvents. Sustainable dominance of suggested methods than the reported HPLC one was ascertained via three user-friendly, trustworthy and up-to-date greenness tools. Additionally, the RGB 12 algorithm, involving freely available Excel sheet, was introduced as a holistic evaluation tool, confirming high adherence to the WAC concept. Clearly, the suggested methods are considered promising alternatives for more sustainable, minimal sample preparation, speedier, and cost saving, for drug assaying in quality control laboratories while maintaining the accuracy, sensitivity, selectivity, and precision of the analytical determinations.

### Electronic supplementary material

Below is the link to the electronic supplementary material.

Supplementary Material 1

## Data Availability

All data generated or analysed during this study is provided within the manuscript or supplementary information files.

## References

[1] Turner RM, DePietro M, Ding B. Overlap of asthma and chronic obstructive pulmonary disease in patients in the United States: analysis of prevalence, features, and subtypes. JMIR Public Health Surveill. 2018;4(8). 10.2196/publichealth.9930 PMC6121140 30126831

[2] Barnes PJ. The cytokine network in asthma and chronic obstructive pulmonary disease. J Clin Invest. 2008;118:3546–56. 18982161 10.1172/JCI36130 PMC2575722

[3] Kässner F, Hodder R, Bateman ED. A Review of Ipratropium Bromide/ Fenoterol Hydrobromide (Berodual ^®^) Delivered Via Respimat ^®^ Soft Mist™ Inhaler in Patients with Asthma and Chronic Obstructive Pulmonary Disease. Vol. 64, Drugs. 2004. 10.2165/00003495-200464150-00005 15257628

[4] Salama RO, Young PM, Rogueda P, Lallement A, Iliev I, Traini D. Advances in drug delivery: is triple therapy the future for the treatment of chronic obstructive pulmonary disease? 12, Expert Opinion on Pharmacotherapy. 2011. p. 1913–32. 10.1517/14656566.2011.589837 21714776

[5] Katsunuma T, Fujita K, Mak JCW, Barnes PJ, Ueno K, Iikura Y. β-Adrenergic agonists and bronchial hyperreactivity: role of β2- adrenergic and tachykinin neurokinin-2 receptors. J Allergy Clin Immunol. 2000;106(1):S104–8. 10887342 10.1067/mai.2000.106636

[6] Jacobson GA, Peterson GM. High-performance liquid chromatographic assay for the simultaneous determination of ipratropium bromide, fenoterol, salbutamol and terbutaline in nebulizer solution. J Pharm Biomed Anal. 1994;12(6):825–32. 7918785 10.1016/0731-7085(94)E0006-M

[7] Bryant DH. Nebulized ipratropium bromide in the treatment of acute asthma. Chest. 1985;88(1). 10.1378/chest.88.1.24 2861067

[8] O’Driscoll BR, Horsley MG, Taylor RJ, Chambers DK, Bernstein A. Nebulized Salbutamol With and Without Ipratropium Bromide in Acute Airflow Obstruction. Lancet. 1989;333(8652). 10.1016/s0140-6736(89)90126-8 2567431

[9] Donohue JF. Combination therapy for chronic obstructive pulmonary disease: Clinical aspects. In: Proceedings of the American Thoracic Society. 2005. pp. 272–81. 10.1513/pats.200505-047SR 16267348

[10] Kawkitinarong K, Ananpipatkul A, Sae-Eao N, Asawawichienchinda T, Sirichana W. Effects of fenoterol/ipratropium bromide on FEV1 and hyperinflation in Thai COPD patients. Chulalongkorn Med J Apr. 2021;65:211–8. 10.58837/CHULA.CMJ.65.2.15

[11] Huhti E, Poukkula A. Comparison of Fenoterol, Ipratropium Bromide, and Their Combination in Patients with Asthma or Chronic Airflow Obstruction. Respiration [Internet]. 1986 Feb 1 [cited 2023 Sep 18];50(Suppl. 2):298–301. 10.1159/000195150 10.1159/000195150 2951825

[12] Ninbovorl J, Sawatdee S, Srichana T. Factors affecting the stability and performance of ipratropium bromide; fenoterol hydrobromide pressurized-metered dose inhalers. AAPS PharmSciTech. 2013;14(4):1294–302. 23975571 10.1208/s12249-013-0024-4 PMC3840779

[13] Anastas P, Eghbali N. Green Chemistry: principles and practice. Chem Soc Rev. 2010;39(1). 10.1039/b918763b 20023854

[14] Inamuddin MA. Green chromatographic techniques: separation and purification of organic and inorganic analytes. Volume 9789400777354. Green Chromatographic Techniques: Separation and Purification of Organic and Inorganic Analytes; 2014.

[15] Fayed AS, Boltia SA, Musaed A, Hegazy MA. Selective quantitation of co-formulated ternary mixture in the presence of potential impurities by liquid chromatographic methods. J Pharm Biomed Anal. 2020;177. 10.1016/j.jpba.2019.112821 31491660

[16] Płotka J, Tobiszewski M, Sulej AM, Kupska M, Górecki T, Namieśnik J. J Chromatogr A. 2013;1307:1–20. Green chromatography. 23932374 10.1016/j.chroma.2013.07.099

[17] Abou Al-Alamein AM, Abd El-Rahman MK, Abdel-Moety EM, Fawaz EM. Green HPTLC-densitometric approach for simultaneous determination and impurity- profiling of ebastine and phenylephrine hydrochloride. Microchem J. 2019;147.

[18] British Pharmacopeia. vol. II, The Stationary Office, London, 2022.

[19] Committee for Medicinal Products for Human Use (CHMP), Guideline on the Pharmaceutical Quality of Inhalation and Nasal Products, in: EMEA/CHMP/QWP/49313/2005. 2006. https://www.ema.europa.eu/en/documents/scientific-guideline/guideline-pharmaceutical-quality-inhalation-and-nasal-products_en.pdf

[20] FDA, Guidance for Industry. (2018). Metered Dose Inhaler (MDI) and Dry Powder Inhaler (DPI) Products-Quality Considerations. US department of health and human services, Food and Drug Administration, Center for Drug Evaluation and Research, 2018. https://www.fda.gov/regulatory-information/search-fda-guidance-documents/metered-dose-inhaler-mdi-and-dry-powder-inhaler-dpi-drug-products-quality-considerations

[21] Marzouk HM, Ibrahim EA, Hegazy MA, Saad SS. Greenness profile assessment of selective liquid chromatographic methods for determination of a quaternary antimigraine combination along with three of their related official impurities. Biomed Chromatogr. 2021;35(9). 10.1002/bmc.5132 33792069

[22] Tantawy MA, Weshahy SA, Wadie M, Rezk MR. Novel HPTLC densitometric methods for determination of tamsulosin HCl and tadalafil in their newly formulated dosage form: comparative study and green profile assessment. Biomed Chromatogr. 2020;34(8). 10.1002/bmc.4850 32302430

[23] Rezk MR, Monir HH, Marzouk HM. Spectrophotometric assessment of the brand new antiviral combination: Sofosbuvir and velpatasvir in their pure forms and pharmaceutical formulation. Spectrochim Acta Mol Biomol Spectrosc. 2019;213. 10.1016/j.saa.2019.01.058 30685554

[24] Variyar PS, Chatterjee S, Sharma A. Fundamentals and theory of HPTLC-Based separation. High-Performance Thin-Layer Chromatography (HPTLC). 2011;27–39. https://link.springer.com/chapter/10.1007/978-3-642-14025-9_2

[25] United States Pharmacopeia and The National Formulary, Rockville MD. USA: USP 44-NF 39, U.S. Pharmacopeial Convention. 2021.

[26] FDA. Validation of chromatographic methods. Center for Drug Evaluation and Research (CDER)., 1994.

[27] ICH harmonized tripartite guideline. Validation of analytical procedures: text and methodology Q2 (R1). 2005.

[28] Stahl M. Peak purity analysis in HPLC and CE using diode-array technology application. Agilent Technol. 2003;8.

[29] Papadoyannis IN, Gika HG. Peak purity determination with a diode array detector. J Liq Chromatogr Relat Technol. 2004;27(6).

[30] Wadie M, Abdel-Moety EM, Rezk MR, Marzouk HM. A novel eco-friendly HPLC method with dual detection modes for versatile quantification of dutasteride and silodosin in pharmaceutical formulation, dissolution testing and spiked human plasma. Microchem J. 2024;197.

[31] Williams RL, Adams WP, Poochikian G, Hauck WW. Content Uniformity and Dose Uniformity: Current Approaches, Statistical Analyses, and Presentation of an Alternative Approach, with Special Reference to Oral Inhalation and Nasal Drug Products. 2002. 10.1023/a:1015114821387 12033365

[32] Pharmacopoeial US, Convention. (905) Uniformity of dosage units. Stage 6 harmonization. 2011.

[33] Armenta S, Garrigues S, de la Guardia M. Green Analytical Chemistry. TrAC - Trends Anal Chem. 2008;27(6):497–511. 10.1016/j.trac.2008.05.003

[34] Larsen C, Lundberg P, Tang S, Ràfols-Ribé J, Sandström A, Mattias Lindh E et al. A tool for identifying green solvents for printed electronics. Nat Commun. 2021;12(1). 10.1038/s41467-021-24761-x PMC8302666 34301943

[35] Alder CM, Hayler JD, Henderson RK, Redman AM, Shukla L, Shuster LE, et al. Updating and further expanding GSK’s solvent sustainability guide. Green Chem. 2016;18(13):3879–90. 10.1039/C6GC00611F

[36] Koller G, Fischer U, Hungerbühler K. Assessing Safety, Health, and Environmental Impact Early during Process Development. Ind Eng Chem Res [Internet]. 2000 [cited 2023 Oct 6];39(4):960–72. 10.1021/ie990669i

[37] Capello C, Fischer U, Hungerbühler K. What is a green solvent? A comprehensive framework for the environmental assessment of solvents. Green Chem. 2007;9(9):927–93. 10.1039/b617536h

[38] Płotka-Wasylka J, Wojnowski W. Complementary green analytical procedure index (ComplexGAPI) and software. Green Chem. 2021;23(21):8657–65. 10.1039/D1GC02318G

[39] Pena-Pereira F, Wojnowski W, Tobiszewski M. AGREE - Analytical GREEnness Metric Approach and Software. Anal Chem. 2020;92(14):10076–82. 32538619 10.1021/acs.analchem.0c01887 PMC7588019

[40] Sharaf YA, Abd El-Fattah MH, El-Sayed HM, Hassan SA. A solvent-free HPLC method for the simultaneous determination of Favipiravir and its hydrolytic degradation product. Sci Rep. 2023;13(1). 10.1038/s41598-023-45618-x PMC10613211 37898682

[41] Nowak PM, Wietecha-Posłuszny R, Pawliszyn J. White Analytical Chemistry: an approach to reconcile the principles of Green Analytical Chemistry and functionality. TrAC - Trends in Analytical Chemistry. Volume 138. Elsevier B.V.; 2021.

[42] Prajapati P, Salunkhe M, Pulusu VS, Shah S. Integrated Approach of White Analytical Chemistry and Analytical Quality by Design to multipurpose RP-HPLC method for synchronous estimation of multiple fixed-dose combinations of paracetamol. Chem Afr. 2024;7(3).

[43] Prajapati P, Patel M, Kansara Y, Shah P, Pulusu VS, Shah S, Green. LC-MS/MS method for in-vivo pharmacokinetics of mirabegron-encapsulated nanostructured lipid carriers in rat plasma: integrating white analytical chemistry and analytical quality by design approach. Sustain Chem Pharm. 2024;39.

[44] Marzouk HM, El-Hanboushy S, Obaydo RH, Fayez YM, Abdelkawy M, Lotfy HM. Sustainable chromatographic quantitation of multi-antihypertensive medications: application on diverse combinations containing hydrochlorothiazide along with LC–MS/MS profiling of potential impurities: greenness and whiteness evaluation. BMC Chem. 2023;17(1). 10.1186/s13065-023-01015-z PMC10439576 37598182

[45] Marzouk HM, Gouda AS, Rezk MR, Abdel-Megied AM. Innovative eco-friendly stability-indicating HPLC-PDA method for simultaneous determination of the emerging antiviral drugs against COVID-19 infection molnupiravir and favipiravir; degradation kinetic studies along with LC-MS based structure elucidation. Microchem J. 2024;205.

