# Supplementary material for: Chromatographic fingerprinting of ipratropium and fenoterol in their novel co-formulated inhaler treating major respiratory disorders; application to delivered dose uniformity testing along with greenness and whiteness assessment
Source: BMC Chem. 2024 Aug 27;18(1):157. doi: 10.1186/s13065-024-01265-5 (PMC11350986; doi:10.1186/s13065-024-01265-5)
Supplement: Supplementary file 1 — Supplementary Material 1 [file 13065_2024_1265_MOESM1_ESM.docx]

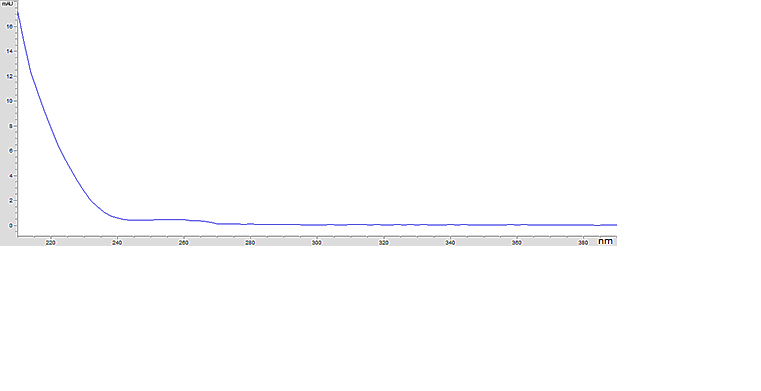


**(a)**


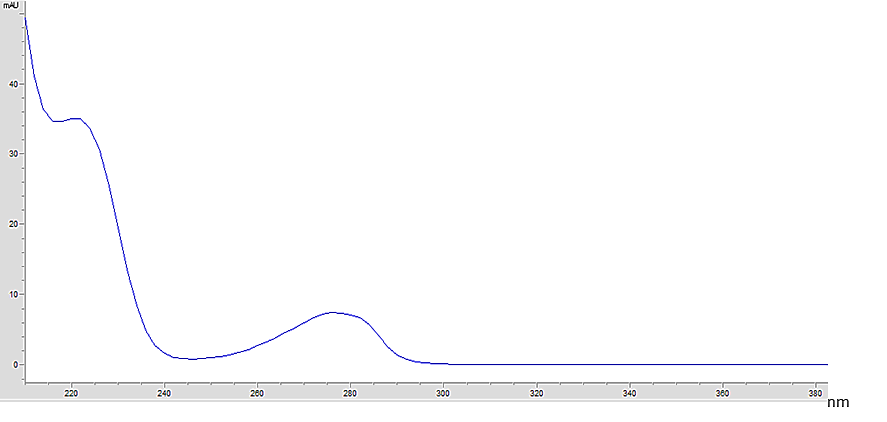


**(b)**


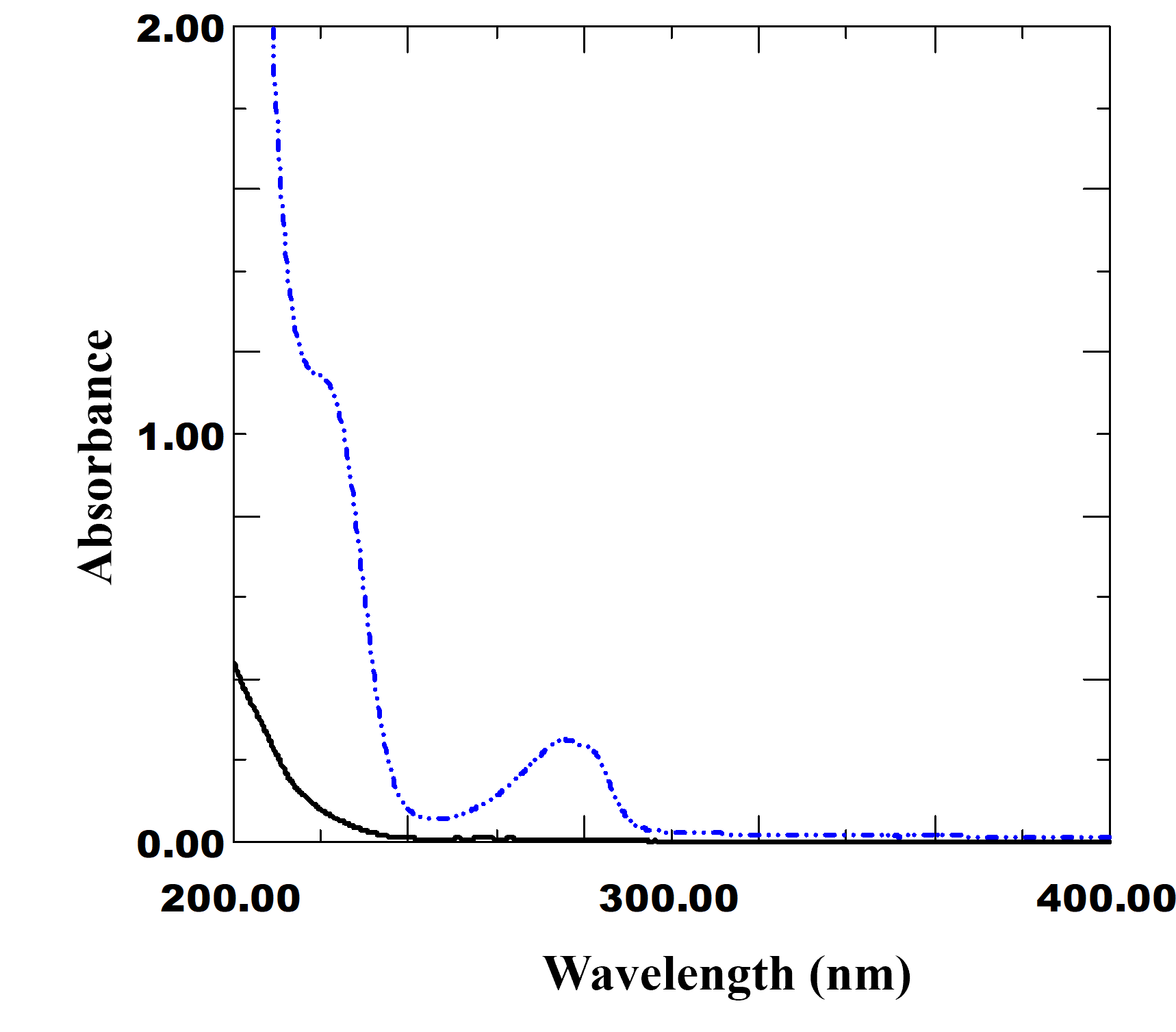


**(c)**

**Fig. S1.**  UV absorption spectra recorded online by DAD detector for (a) Ipratropium and (b) Fenoterol peaks in the optimized mobile phase, and (c) Overlaid zero-order absorption spectra of Ipratropium (**―**) and Fenoterol (**….**) recorded by off-line UV spectrophotometry.


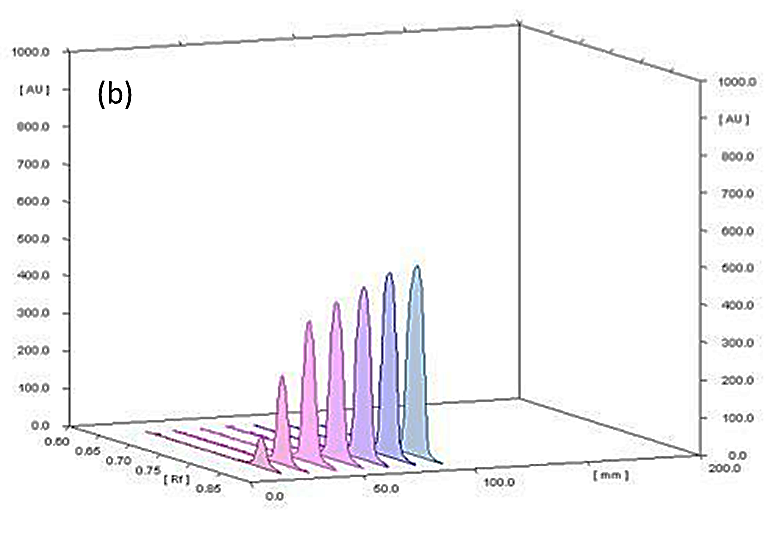

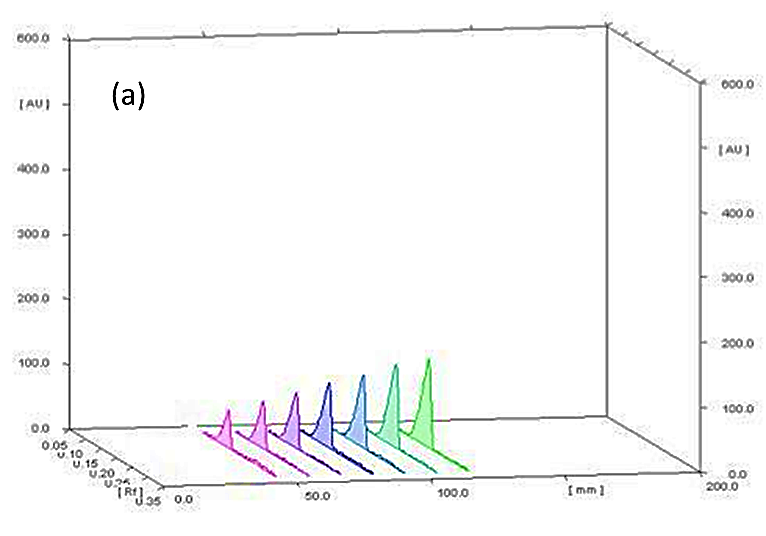


**Fig. S2.** Scanning 3D profiles of (a) IPR (0.5 – 15.0 μg/band) and (b) FEN (0.5 – 12.0 μg/band).

**
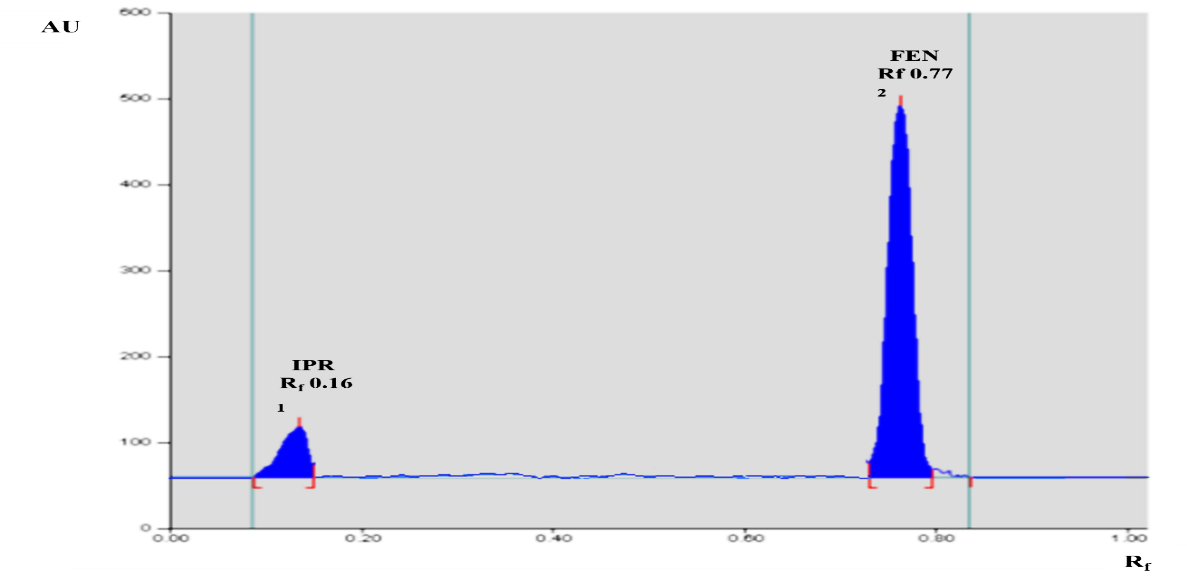
**

**(a)**

**
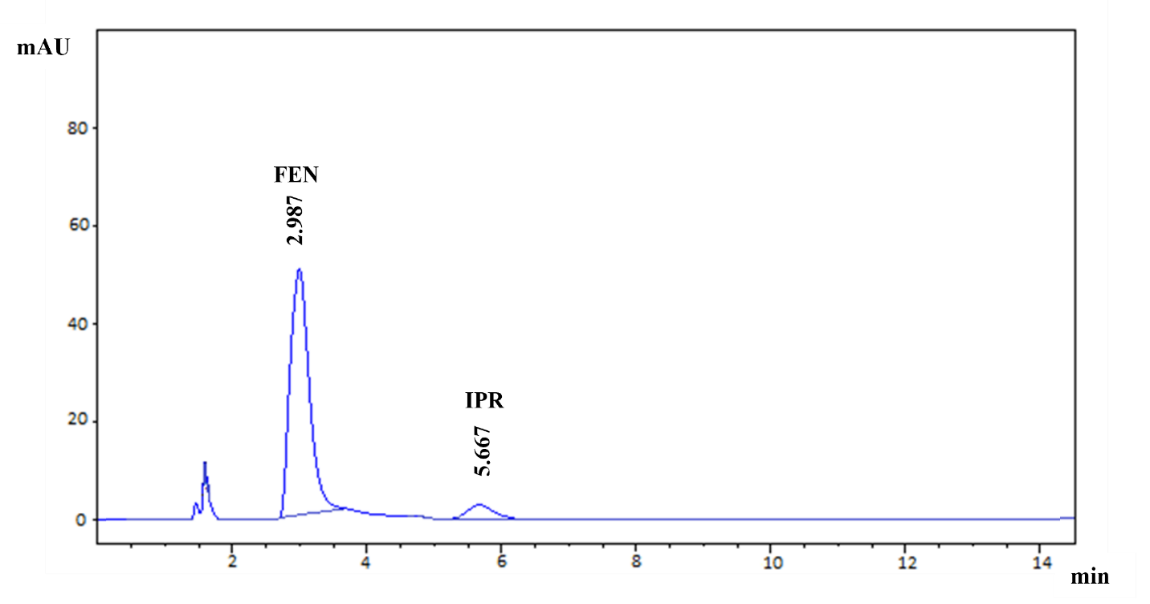
**

**(b)**

**Fig. S3.** **(a)** HPTLC densitogram of IPR (1.6 μg/band) and FEN (4.0 μg/band) and **(b)** HPLC-DAD chromatogram of IPR (20.0 μg/mL) and FEN (50.0 μg/mL) in Atrovent^®^ comp HFA inhaler.

**(a)**


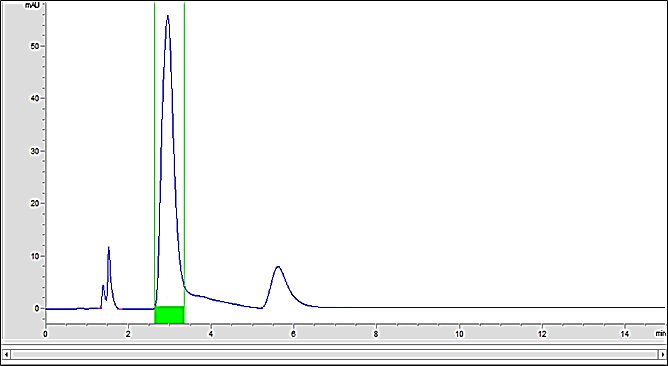

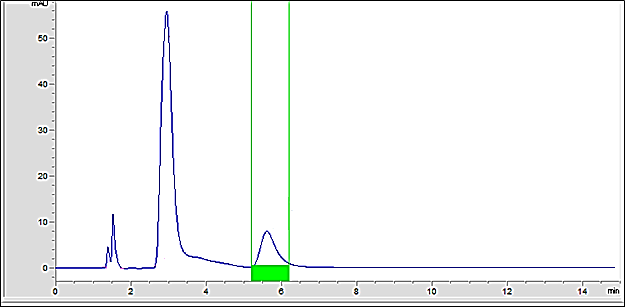

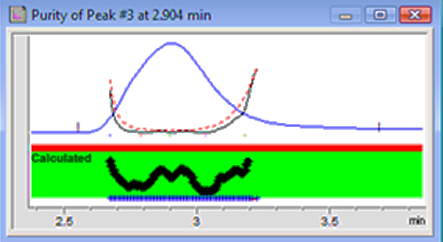

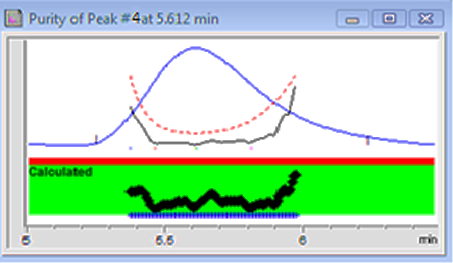


**(b)**

**Fig. S4.** Peak purity assessment for (a) Fenoterol peak at t_R_ = 2.90 min (± 0.1) with purity factor of 999.973 and (b) Ipratropium peak at t_R_ = 5.61 min (± 0.1) with purity factor of 999.934 manipulated at peak threshold of 990.

**
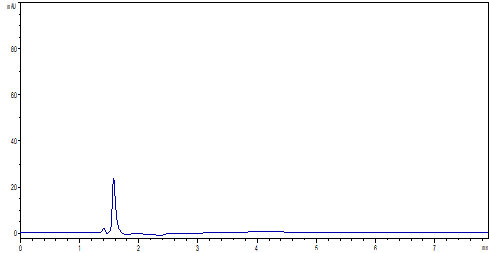
**

**Fig. S5.** High performance liquid chromatogram of blank sample.

| Mixture  No. | Claimed concentration (µg/band) | |  | Recovery % | | |  | Claimed concentration (µg/mL) | |  | Recovery % | |
| --- | --- | --- | --- | --- | --- | --- | --- | --- | --- | --- | --- | --- |
|  |  |  |  | **HPTLC-densitometry** | | |  |  |  |  | **HPLC-DAD** | |
|  | IPR | FEN |  | IPR | FEN | |  | IPR | FEN |  | IPR | FEN |
| 1 | 2 | 2 |  | 101.27 | 100.95 | |  | 20 | 20 |  | 101.86 | 100.87 |
| 2 | 2 | 4 |  | 100.15 | 101.74 | |  | 20 | 40 |  | 100.66 | 101.38 |
| 3* | 2 | 5 |  | 98.40 | 99.83 | |  | 20 | 50 |  | 98.91 | 101.07 |
| 4 | 2 | 6 |  | 101.21 | 99.99 | |  | 20 | 60 |  | 99.41 | 100.75 |
| 5 | 4 | 2 |  | 99.07 | 101.96 | |  | 40 | 20 |  | 100.22 | 101.30 |
| 6 | 6 | 2 |  | 100.81 | 99.96 | |  | 60 | 20 |  | 98.62 | 101.06 |
|  | Mean  ± RSD% | |  | 100.15 ±1.185 | | 100.74 ± 0.945 |  | Mean  ± RSD% | |  | 99.95 ± 1.214 | 101.07 ± 0.239 |

**Table S1.** Determination of Ipratropium and Fenoterol in laboratory prepared mixtures by the proposed HPTLC-densitometry and HPLC-DAD methods.

*Laboratory mixture prepared at the ratio of dosage form.

**Table S2.** Comparative statistical analysis of the results obtained by the proposed chromatographic methods and the official ones for determination of pure Ipratropium and Fenoterol.

| **Parameter** | **HPTLC-densitometry**  **IPR FEN** | | **HPLC-DAD**  **IPR FEN** | | **Official methods**  **IPR ^a^ FEN ^b^** | |
| --- | --- | --- | --- | --- | --- | --- |
| **Mean of recoveries** | 100.13 | 99.15 | 99.85 | 100.10 | 99.40 | 99.40 |
| **SD** | 1.610 | 1.205 | 1.216 | 0.787 | 0.926 | 1.062 |
| **Variance** | 2.592 | 1.452 | 1.479 | 0.619 | 0.857 | 1.128 |
| **n** | 6 | 6 | 6 | 6 | 6 | 6 |
| **Student’s t-test (2.228) ^c^** | 0.963 | 0.381 | 0.721 | 1.297 | - | - |
| **F-test (5.05)^c^** | 3.024 | 1.287 | 1.726 | 1.822 | - | - |

^a^ IPR is determined by potentiometric titration method using 0.01 M silver nitrate as titrant with potentiometric detection of end point as per the British Pharmacopoeia **[18]**.

^b^ FEN is determined by titrimetric method using 0.1 M ammonium thiocyanate as titrant and ferric alum as indicator until an orange color is obtained as per the British Pharmacopoeia **[18]**.

^c^ The values in parentheses represent the corresponding tabulated values of t and F at p=0.05.

**Table S3.** Comparison between the proposed HPTLC-densitometry and HPLC-DAD methods for analysis of IPR and FEN in Atrovent^®^ comp HFA inhaler, and the reported HPLC method.

| **Parameters** | **Proposed HPTLC - densitometry** | **Proposed HPLC - DAD** | **Reported HPLC Method [6]** |
| --- | --- | --- | --- |
| **Mobile phase** | Ethyl acetate-ethanol- acetic acid (5.0:5.0:0.1, by volume) | 10.0 mM phosphate buffer (pH 5.0) – methanol (70:30, v/v) | Ternary gradient analysis (A, B and C):  A: tetrahydrofuran-distilled water (40:60, v/v) containing 0.0025 M ion pair reagent,  B: distilled water, and C: methanol-distilled water (50:50, v/v) |
| **Stationary phase** | Aluminum sheets (20 × 10 cm) pre-coated with silica gel 60 F_254_ | Zorbax SB C_18_ column  (150 × 4.6 mm, 5.0 µm) | Nova-Pak C_18_ column  (100 × 8 mm, 4 µm) |
| **Flow rate** | - | 1.0 mL/min | 2.0 mL/min |
| **Wavelength** | 220.0 nm | 220.0 nm | 220.0 nm |
| **Linearity** | IPR: 0.50–15.0 µg/band  FEN: 0.50–12.0 µg/band | 5.0–200.0 µg/mL for both drugs | IPR: 20.8-250.0 µg/mL  FEN: 27.8-500.0 µg/mL |
| **Run time** | - | 7 min | 13 min |
| **Applications and merits** | The first simple HPTLC-densitometric method for simultaneous determination of IPR and FEN in their combined metered dose inhaler with greenness and whiteness assessment | The first simple HPLC-DAD method for simultaneous determination of IPR and FEN in their combined metered dose inhaler and delivered dose uniformity testing with greenness and whiteness assessment. | Assessment of IPR and FEN, separately, along with other drugs in various “single” nebulizer solutions as well as stability study. |
